# Continued disruptions in health care services and mental health among health care providers during the COVID-19 pandemic in five sub-Saharan African countries

**DOI:** 10.7189/jogh.12.05046

**Published:** 2022-11-12

**Authors:** Nega Assefa, Yasir Younis Abdullahi, Elena Cori Hemler, Bruno Lankoande, Dongqing Wang, Isabel Madzorera, Ourohiré Millogo, Livesy Naafoe Abokyi, Dielbeogo Dasmane, Kassoum Dianou, Angela Chukwu, Firehiwot Workneh, Frank Mapendo, Abbas Ismail, Sulemana Watara Abubakari, Emily Smith, Ayo Oduola, Abdramane Soura, Ali Sie, Japhet Killewo, Mary Mwanyika-Sando, Said Ali Hamad Vuai, Till Baernighausen, Kwaku Poku Asante, Tajudeen Raji, Yemane Berhane, Wafaie Wahib Fawzi

**Affiliations:** 1 College of Health and Medical Sciences, Haramaya University, Harar, Ethiopia; 2 Department of Global Health and Population, Harvard T.H. Chan School of Public Health, Harvard University, Boston, Massachusetts, United States of America; 3 Institut Supérieur des Sciences de la Population, University of Ouagadougou, Ouagadougou, Burkina Faso; 4 Nouna Health Research Center, Burkina Faso; 5 Kintampo Health Research Center, Kintampo, Research and Development Division, Ghana Health Service, Kintampo North Municipality, Bono East Region, Ghana; 6 Department of Health Services, Policy, Planning, Management and Economics, School of Public Health, University for Development Studies, Tamale, Ghana; 7 Department of Statistics, University of Ibadan, Ibadan, Nigeria; 8 Addis Continental Institute of Public Health, Ethiopia; 9 Africa Academy for Public Health, Dar es Salaam, Tanzania; 10 College of Natural and Mathematical Sciences, University of Dodoma, Dodoma, Tanzania; 11 Department of Global Health, Milken Institute School of Public Health, George Washington University, Washington, DC, United States of America; 12 Department of Exercise and Nutrition Sciences, Milken Institute School of Public Health, George Washington University, Washington, DC, United States of America; 13 University of Ibadan Research Foundation, University of Ibadan, Ibadan, Nigeria; 14 Department of Epidemiology and Biostatistics, Muhimbili University of Health and Allied Sciences, Dar es Salaam, Tanzania; 15 Heidelberg Institute of Global Health, University of Heidelberg, Heidelberg, Germany; 16 Africa Health Research Institute, KwaZulu-Natal, South Africa; 17 Division of Public Health Institutes and Research, Africa Centres for Disease Control and Prevention, Addis Ababa, Ethiopia; 18 Department of Nutrition, Harvard T.H. Chan School of Public Health, Harvard University, Boston, Massachusetts, United States of America; 19 Department of Epidemiology, Harvard T.H. Chan School of Public Health, Harvard University, Boston, Massachusetts, United States of America

## Abstract

**Background:**

Continuous monitoring of the pandemic’s impact on health service provision and mental health, COVID-19 perceptions, and compliance with prevention measures among health care providers (HCPs) can help with mitigating the pandemic’s negative effects.

**Methods:**

A computer-assisted telephone interviewing (CATI) survey was conducted among 1499 HCPs in Burkina Faso (Ouagadougou), Ethiopia (Addis Ababa), Nigeria (Lagos and Ibadan), Tanzania (Dar es Salaam), and Ghana (Kintampo). Self-reported mental health, perceptions of the COVID-19 pandemic, and prevention measures available in the workplace were assessed. HCPs’ responses to questions regarding the impact of COVID-19 on nine essential health services were summed into a score; high service disruption was defined as a score higher than the total average score across all sites. Modified Poisson regression was used to identify potential factors related to high service disruption.

**Results:**

Overall, 26.9% of HCPs reported high service disruption, with considerable differences across sites (from 1.6% in Dar es Salaam to 45.0% in Addis Ababa). A considerable proportion of HCPs reported experiencing mild psychological distress (9.4%), anxiety (8.0%), and social avoidance or rejection (13.9%) due to their profession. Participants in Addis Ababa (absolute risk ratio (ARR) = 2.10; 95% confidence interval (CI) = 1.59-2.74), Lagos (ARR = 1.65; 95% CI = 1.24-2.17), and Kintampo (ARR = 2.61; 95% CI = 1.94-3.52) had a higher likelihood of reporting high service disruption compared to those in Ouagadougou. Reporting ever-testing for COVID-19 (ARR = 0.82; 95% CI = 0.69-0.97) and the presence of COVID-19 guidelines in the workplace (ARR = 0.63; 95% CI = 0.53-0.77) were both associated with lower reported health service disruption among HCPs.

**Conclusion:**

The COVID-19 pandemic continues to disrupt essential health services and present a challenge to HCPs’ mental health, with important differences across countries and settings; interventions are needed to mitigate these negative effects of the pandemic.

Coronavirus disease 2019 (COVID-19) is a severe acute respiratory syndrome that was first reported in December 2019 [1,2] and has become a global public health emergency of international concern and a pandemic [3]. This highly infectious illness has claimed more than six million lives globally, including a quarter million on the African continent [4]. Despite the introduction of several preventive strategies including the development and emergency use of vaccines, the pandemic continues to strain and impact health care systems and health care providers (HCPs), especially those in sub-Saharan Africa (SSA) [5,6].

The World Health Organization (WHO) estimated that between 80 000 and 180 000 HCPs died globally due to COVID-19 from January 2020 to May 2021. As of November 2021, 27% of HCPs in the African region had been vaccinated, with large differences across countries. Between March-November 2021, 1.3 million HCPs were fully vaccinated across 25 countries in Africa. Six countries had fully vaccinated more than 90% of HCPs, while nine countries reached less than 40%, compared to 80% among 22 mostly high-income countries [7]. HCPs are at the frontlines of the COVID-19 pandemic treating and caring for infected patients, which has made them the most vulnerable to pathogen exposure, burnout, fatigue, and psychological stress [5,8].

To help control the virus’s spread, it is important to understand perceptions regarding COVID-19 and preventive practices among HCPs [9]. Previous reports have shown that HCPs in sub-Saharan Africa (SSA) had incorrect perceptions and low COVID-19 preventive practices, despite high levels of knowledge [10]. As the fight to control the pandemic continues, HCP’s anxiety, sleep disturbance, substance abuse, and burn-out may exacerbate the burden of depressive and anxiety disorders among HCPs [11,12]. It is also important to monitor changes in COVID-19 perceptions and compliance with prevention measures, as well as address mental health problems, including social stigma towards HCPs, to better ensure their safety and prepare them to deliver health services [13].

Moreover, efforts to limit the spread of the virus often affect the delivery of essential health services, especially in resource-limited countries [14]. A recent study on the impacts of COVID-19 in SSA in 2020 found that more than half of essential health services were disrupted because of COVID-19 [13]. Consequently, African Center for Disease Control and Prevention (CDC) released a guidance document for African Union Member States on actions to be taken to ensure they continue to meet all the health needs of their citizens [15].

This study is a follow-up to a baseline cross-sectional study conducted in 2020 among HCPs in Nigeria, Ethiopia, and Burkina Faso; this second survey round was conducted to re-assess mental health; COVID-19 preventive measures and perceptions; and disruption of health services from the perspective of HCPs during the COVID-19 pandemic and examine changes over time. Evidence on these areas of concern is lacking among HCPs in SSA. Data on HCP’s experiences during the COVID-19 pandemic that is comparable over time is needed to help policymakers in SSA identify COVID-19 impacts and prioritize areas of intervention. In this second-round survey, we hypothesize that compliance with preventive measures, mental health disruptions, and level of reported health care interruptions among HCPs may have decreased compared to the earlier survey. This second survey round also included new sites in Tanzania and Ghana, providing much-needed evidence on HCP’s COVID-19 experiences in these diverse contexts. Evidence from this study is critical for investigating the determinants of health service disruption in sub-Saharan Africa and monitoring trends in preventive practices, perceptions, and mental health among HCPs during the COVID-19 pandemic.

## METHODS

### Study setting and design

This survey was the second round of an ongoing study conducted by the Africa Research, Implementation Science, and Education (ARISE) Network, a platform for research and training which includes 21 member institutions from nine sub-Saharan African countries. This survey utilized computer-assisted telephone interviewing (CATI) to assess the impacts of the COVID-19 pandemic and vaccine hesitancy among adolescents, adults, and HCPs in SSA. The first round of the survey was conducted in Ethiopia, Nigeria, and Burkina Faso in 2020 among HCPs, adults, and adolescents. A second survey round was conducted in 2021, approximately one year after the first survey round, among the same countries and expanding to new sites in Ghana and Tanzania.

The current study focuses on data collected among HCPs in the second survey round regarding perceptions about COVID-19, preventive practices, mental health, and their reported disruption of health care service provision. Data on adults and adolescents will be published separately. This second round survey collected data from HCPs in major urban areas in each country, namely Ouagadougou (Burkina Faso), Addis Ababa (Ethiopia), Lagos, Ibadan (Nigeria), and Dar es Salaam (Tanzania). Data were also collected from Ghana (Kintampo), which is a largely rural area. The study included approximately 300 HCPs in each country and 1499 in total.

The round 2 survey took place from July to December 2021. The study rationale, sampling methods, descriptions of the study sites in Ethiopia, Nigeria, and Burkina Faso, and the use of CATI to conduct the phone interviews are described in detail elsewhere [16]. Briefly, the telephone numbers for the HCPs were accessed through public repositories, professional associations, and health care facilities. HCPs were eligible to be included in the study if they were currently working in a health care setting; dentists, pharmacists, and other allied health professionals were excluded. HCPs from both public and private health facilities were recruited with no restrictions on medical specialty or whether they provided COVID-19-related services. Participants included in the round one survey in Ethiopia, Burkina Faso and Nigeria were re-contacted, asked if they would like to participate in the round two survey and re-consented. Those who declined to participate were replaced with new participants in each country. The details of the methods for the second-round survey can be found online elsewhere [17].

Study sites in Tanzania and Ghana were added during the second survey, so all participants from these sites had not participated in the first survey round. In Tanzania, participants were sampled from Dar es Salaam, the largest city in Tanzania and the country’s financial and business centre. Lists including names and telephone numbers of 338 nurses and 384 physicians/doctors were obtained from health care facilities in Dar es Salaam through regional and local government authorities. From each list, 160 nurses and 175 physicians/doctors were sampled and contacted via phone calls. Due to challenges in reaching health care providers over the phone and completing interviews, it was necessary to set up phone appointments and visit HCPs to conduct interviews face to face. To reach the final sample size of 300 health care providers, face-to-face interviews were conducted in 19 health care facilities.

**Figure 1:**
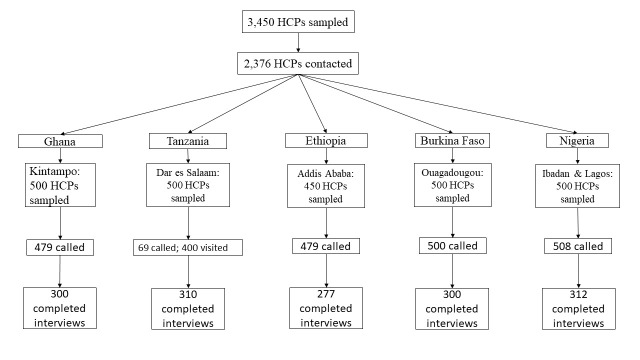
ARISE COVID-19 Survey Round 2 participant flowchart for the health care providers survey across five countries, 2021.

The names and contact numbers of 1783 HCPs in Ghana were obtained from Ghana Health Service Health Directorates and some private health facilities within the Kintampo Health and Demographic Surveillance area (which includes the mainly rural communities of 6 adjoining administrative areas). A total of 500 health care workers were randomly sampled to participate in the survey and 479 health care workers were called to obtain the target sample size of 300 health care workers (**Figure 1**).

This survey was approved by ethical review boards at Harvard T.H. Chan School of Public Health (IRB20-0909) and ethical committees in each country, including Nouna Health Research Center Ethical Committee (2020-009-/MS/SG/INSP/ CRSN /CIE) and National Ethics Committee in Burkina Faso (2020-7-127); the Institutional Ethical Review Board of Addis Continental Institute of Public Health in Ethiopia; the University of Ibadan Research Ethics Committee in Nigeria; Muhimbili University of Health and Allied Sciences (Ref. No. DA 282/298/06/C/767), University of Dodoma (Ref. No. MA.84/261/02/134) and National Institute of Medical Research in Tanzania (Ref. No. NIMR/HQ/R.8a/Vol. IX/3775); and the Kintampo Health Research Centre Institutional Ethics Committee in Ghana. The study also conformed to the principles embodied in the Declaration of Helsinki [18,19].

### Data collection

All data were collected by trained enumerators using CATI, except for Tanzania, where interviews were conducted face to face due to challenges in reaching HCPs over the phone. All research teams were trained on study protocols, including screening, consent, enrollment, and data collection, emphasizing the safeguarding of the study participants' rights and well-being and maintaining confidentiality. The consent script and study instruments were translated into the local languages of each country and piloted before use in each site. The data collectors obtained informed consent electronically and verbally and used standardized telephone scripts when communicating with the participants.

### Statistical analysis

Sociodemographic characteristics, COVID-19 perceptions, preventive practices, mental health, and disruption of health care services from the perspective of HCPs were reported descriptively. For the descriptive analyses, we calculated means and standard deviations (SDs) overall and by country for normally distributed continuous variables, medians, 25th percentiles (Q1), and 75th percentiles (Q3) for skewed continuous variables, and counts and percentages for categorical variables. We did not conduct analyses across rounds 1 and 2 due to the low retention rate of HCPs in Nigeria (47%) and the inclusion of additional sites in Tanzania and Ghana in the second survey round.

The HCP questionnaire included two questions assessing the providers’ perceptions of COVID-19, ie, whether they were concerned about the spread of COVID-19 and their level of perceived risk of exposure to COVID-19. It also included questions assessing preventive measures available in the workplace from the perspective of the HCPs, whether their workplace had formulated COVID-19 policies and guidelines and whether they had received formal training or orientation on COVID-19 in the workplace in the last six months. HCPs also responded to questions regarding the type of COVID-19 treatment available in the facility, the type of COVID-19 testing available, whether the testing was free or paid, whether they had ever been tested or tested positive for COVID-19, and whether they had treated COVID-19 patients. The full questionnaire is available elsewhere [17].

HCPs’ mental health over the prior two weeks was assessed using the four-item Patient Health Questionnaire for Depression and Anxiety Scale (PHQ-4), a validated instrument for screening for anxiety and depression that has been used previously in sub-Saharan Africa [20,21]. To assess anxiety, participants were asked how often they had been bothered by the following problems over the past two weeks: “Feeling nervous, anxious or on edge” and “Not being able to stop or control worrying”, with response options coded as 0 for “Not at all,” 1 for “Several days,” 2 for “More than half the days,” and 3 for “Nearly every day.” To assess depression, the prompts “Feeling down, depressed or hopeless,” and “Little interest or pleasure in doing things” were asked using the same answer options as the anxiety questions. Anxiety and depression subscales were created by summing the two questions specific to anxiety and the two questions specific to depression, each with a range of 0-6. For each subscale, a score of three or above was considered positive for anxiety and depression screening purposes [13,22].

All PHQ-4 items were summed into a scale to measure psychological distress, with a total score from zero to twelve. Scores of two, five, eight, and nine were the cut-off points for none, mild, moderate, and severe psychological stress, respectively. We also assessed perceived stigma by asking whether the HCPs had experienced 1) congratulations or acknowledgment, 2) social avoidance or rejection, 3) denial of health care, education, housing, or employment, and 4) physical violence due to their profession in the last six months.

HCPs were also asked how they perceived COVID-19 currently impacting nine specific health services. For each service, participants had the option to respond with no impact, services are paused, services are operating at minimum level/only provided for emergencies, don’t know/don’t work in the unit, not applicable/service not available at facility, and refuse to answer. Responses were scored as 0 if there was no interruption (no impact on services), 1 for partial interruption (services operating at a minimum level/emergency services provided only), and 2 for complete interruption (services are paused). HCPs with missing responses and those who refused to answer, did not know/did not work in the unit, or answered not applicable were excluded from the analysis. The specific health services included in the questionnaire were grouped into child health services (including childhood immunization, vitamin A supplementation for children, management of child malnutrition), maternal and reproductive health services (including antenatal care for pregnant women, iron and folic acid for pregnant women, and sexual and reproductive health including family planning), and other health care services (HIV and tuberculosis treatment services, surgeries, and outpatient services.) For each category of health service (child health services, maternal and reproductive health services, and other health care services), an aggregated score was computed by summing HCP’s responses to each question included in each specific health service (ranging from 0-6 for each health service). A total service interruption score was created by summing the scores for the nine individual services. The total interruption score was defined as high using the average aggregated score as a cut-off across all sites [23,24].

The binary outcome of a high interruption score was used as the outcome for analyses examining determinants of health services disruption (1 if the total mean aggregated score was >1.81 and not missing, and 0 if the score was ≤1.81). Potential determinants included age of the HCP, gender, type of health care facility (government hospital/facility, private hospital/facility, and health outposts), occupation (doctors and nurses and others), country of residence, COVID-19 testing availability in their workplace, if they had ever been tested for COVID-19, their perception on COVID-19 spread and risk of infection, availability of workplace guidelines regarding COVID-19, and presence of mild psychological distress. These were selected based on the World Health Organization (WHO) health systems contextual framework, which is categorized into four themes (governance, guidance, mitigation actions, and information for decision-making) for maintaining provision and use of health services during the COVID-19 pandemic. Modified Poisson regression [25,26] was used to calculate crude risk ratios (CRRs) and adjusted risk ratios (ARRs) with 95% confidence intervals (CIs). Tanzania was excluded from the models to enable model convergence due to the very small proportion of HCPs reporting high service disruption (1.7%). Factors with a *P* value of ≤0.25 in the crude analysis were retained for the final model. HCP gender was added to the final model, irrespective of the *P* value in the crude analysis because the authors thought it was important in adjusting the model. Data were cleaned and managed using SAS 9.4 (SAS Institute Inc., Cary, North Carolina) and analyzed using Stata version 16 (StataCorp LLC, College Station, Texas) at a two-sided α level of 0.05. Missing data were handled using a complete case analysis.

## RESULTS

### Sociodemographic characteristics of the health care providers

A total of 1499 HCPs agreed to participate in the survey, including about 300 from each country. The majority of the HCPs in Burkina Faso (74.0%) and Ethiopia (75.1%) participated in the round one survey, whereas only 47.1% in Nigeria did (**Table 1**). Most of the study population worked in governmental health institutions (66.1%) as opposed to private institutions (15.3%) and health outposts (18.6%), and the majority were nurses (65.6%) as opposed to doctors (27.4%). Participants' median age was 38, 30, 40, 32, and 29 years for Burkina Faso, Ethiopia, Nigeria, Tanzania, and Ghana, respectively. Female HCPs accounted for the majority of those surveyed in Tanzania (69.0%), Nigeria (69.5%), Ethiopia (59.2%), and Ghana (54.0%) but for less than half of those surveyed in Burkina Faso (46.0%).

**Table 1:** Sociodemographic and other characteristics of health care providers in a phone-based survey in five sub-Saharan African countries, 2021*

	Burkina Faso	Ethiopia	Nigeria	Tanzania	Ghana	
	**Ouagadougou**	**Addis Ababa**	**Ibadan and Lagos**	**Dar es Salaam**	**Kintampo**	**Total**
**Number of health care providers included in the survey**	300	277	312	310	300	1499
**Number of health care providers who also participated in round 1 survey**	222 (74.0)	208 (75.1)	147 (47.1)	NA†	NA†	577 (64.9)
**Age (years)‡**	38.0 (32, 46)	30.0 (28, 36)	40.0 (30, 47.5)	32.0 (28, 40)	29.0 (28, 33)	35.8 (28, 41)
**Female**	138 (46.0)	164 (59.2)	217 (69.5)	214 (69.0)	164 (54.7)	897 (59.8)
**Type of health facility**						
Governmental hospital/clinic	207 (69.3)	207 (74.7)	224 (71.8)	182 (58.7)	169 (56.3)	990 (66.1)
Private hospital/clinic	87 (29.0)	51 (18.4)	80 (25.6)	0 (0)	12 (4.0)	230 (15.3)
Health outposts and other	5 (1.7)	19 (7.0)	8 (2.6)	127 (41.3)	119 (39.7)	279 (18.6)
**Occupation**						
Doctor	85 (28.3)	118 (42.6))	99 (31.7)	105 (33.9)	3 (1.0)	410 (27.4)
Nurse	197 (65.7)	157 (56.7)	182 (58.3)	179 (57.7)	269 (89.7)	984 (65.6)
Others§	18 (6.0)	2 (0.7)	31 (10.0)	26 (8.4)	28 (9.3)	105 (7.0)
**Religion**						
Catholic	149 (49.7)	3 (1.1)	23 (7.4)	79 (25.5)	70 (23.3)	324 (21.6)
Muslim	106 (35.3)	21 (7.5)	41 (13.1)	78 (25.2)	40 (13.3)	286 (19.1)
Orthodox Christian	3 (1.0)	198 (71.5)	141 (45.2)	1 (0.3)	37 (12.3)	380 (25.3)
Protestant	39 (13.0)	52 (18.8)	87 (27.9)	149 (48.0)	152 (50.7)	479 (32.0)
Other	3 (1.0)	3 (1.1)	20 (6.4)	3 (1.0)	1 (0.3)	30 (2.00)

NA – not applicable

*All data are presented as the number of observations (percentage) unless otherwise specified.

†Countries did not participate in the round one survey.

‡Median (25th and 75th percentile).

§Clinical officers and community health workers.

### Perceptions of COVID-19 and preventive measures among health care providers

Almost all participants in Ghana (99.7%), Nigeria (97.1%), and Ethiopia (96.7%), and most in Burkina Faso (92.0%) and Tanzania (83.0%) were concerned about the spread of COVID-19 (**Table 2**). Most HCPs (≥80%) in Ethiopia, Nigeria, and Tanzania perceived high or very high risks of contracting COVID-19, but only 58.0% of those in Burkina Faso did. Nearly all participants reported that their workplaces implemented prevention measures, including wearing masks and hand washing. While HCPs reported that using personal protective equipment (PPE) was common in Burkina Faso (91.3%), Ethiopia (83.4%), and Nigeria (74.0%), it was less common in Tanzania (61.9%) and Ghana (55.0%). Less than half (43.7%) of the HCPs in Burkina Faso reported implementing social distancing between patients.

**Table 2:** Perceptions and preventive practices regarding COVID-19 among health care providers in a phone-based survey in five sub-Saharan African countries, 2021*

	Burkina Faso	Ethiopia	Nigeria	Tanzania	Ghana	
	**Ouagadougou**	**Addis Ababa**	**Ibadan and Lagos**	**Dar es Salaam**	**Kintampo**	**Total**
**Number of health care providers**	300	277	312	310	300	1499
**Concerned about the spread of COVID-19**	276 (92.0)	268 (96.7)	303 (97.1)	257 (83.0)	299 (99.7)	1403(93.6)
**Perceived level of risk of COVID-19 exposure**						
No risk	4 (1.3)	1 (0.4)	2 (0.6)	12 (3.9)	4 (1.3)	23 (1.5)
Low risk	122 (40.7)	36 (13.0)	29 (9.3)	29 (9.3)	28 (9.3)	244 (16.3)
High risk	140 (46.7)	136 (49.1)	164 (52.6)	188 (60.7)	176(58.7)	804 (53.6)
Very high risk	34 (11.3)	104 (37.5)	117 (37.5)	81 (26.1)	92 (30.7)	428 (28.6)
**Preventive measures implemented in the workplace**						
Wearing masks	295 (98.3)	275 (99.3)	311 (99.7)	279 (90.0)	298 (99.3)	1458(97.3)
Using personal protective equipment	274 (91.3)	231(83.4)	231 (74.0)	192 (61.9)	165 (55.0)	1093 (72.9)
Hand washing	296 (98.7)	258 (93.1)	309 (99.0)	299 (96.4)	300 (100)	1462 (97.5)
Keeping sufficient distance between patients	131 (43.7)	183 (66.1)	280 (89.7)	203 (65.5)	243 (81.0)	1040 (69.4)
Presence of sanitizers/ hand washing station	268 (89.3)	208 (75.1)	301 (96.5)	303 (97.7)	293 (97.7)	1373 (91.6)
Regular cleaning/decontamination	268 (89.3)	220 (79.4)	286 (91.7)	241 (77.7)	292 (97.3)	1307 (87.2)
Temperature checks	257 (85.6)	154 (55.6)	279 (89.4)	208 (67.1)	288 (96.0)	1186 (79.1)
Workplace formulated COVID-19 guidelines/policies	180 (60.0)	208 (75.1)	297 (95.2)	208 (67.1)	269 (89.7)	1162 (77.5)
**COVID-19 formal training/orientation in the workplace in the past 6 months**						
Natural course	35 (11.7)	90 (32.5)	195 (62.5)	78 (25.2)	163 (54.3)	561(37.4)
Management and treatment	31 (10.4)	89 (32.1)	183 (58.7)	72 (23.2)	133 (44.3)	508 (33.9)
How vaccines work	20 (6.7)	81 (29.2)	160 (51.3)	74 (23.9)	105 (35.0)	440 (29.3)
Managing vaccination programs	36 (12.0)	77 (27.8)	112 (35.9)	66 (21.3)	52 (17.3)	343 (22.9)
**Type of COVID-19 treatment available in facility**						
Corticosteroids	31 (10.3)	129 (46.6)	54 (17.3)	90 (29.0)	13 (4.3)	317 (21.1)
Remdesivir	3 (1.0)	73 (26.4)	31 (9.9)	11 (3.5)	0 (0)	118 (7.9)
Antibiotics	131 (43.7)	174 (62.8)	136 (43.6)	176 (56.8)	91 (30.3)	708 (47.2)
Ivermectin	5 (1.68)	47 (17.0)	91 (29.2)	13 (4.2)	1 (0.3)	157 (10.5)
Chloroquine	116 (38.7)	28 (10.1)	18 (5.8)-	6 (1.9)	18 (6.0)	186 (12.4)
Multivitamin	42 (14.0)	82 (29.6)	141 (45.2)	139 (44.5)	96 (32.0)	500 (33.4)
**COVID-19 testing available at the workplace**	175 (58.3)	240 (86.6)	205 (65.7)	111 (35.8)	112 (37.3)	843 (56.2)
If yes, free testing available	154 (88.0)	209 (87.1)	154 (75.1)	40 (36.0)	85 (75.9)	642 (76.2)
If yes, type of COVID-19 test available†						
PCR	108 (61.7)	118 (50.2)	124 (60.5)	24 (21.4)	46 (41.1)	420 (50.1)
Antigen	90 (51.4)	134 (56.3)	85 (41.5)	55 (49.1)	17 (15.2)	381 (45.2)
**Ever been tested for COVID-19**	143 (47.7)	216 (78.0)	169 (54.2)	42 (13.5)	128 (42.8)	698 (46.6)
**Tested positive for COVID-19**	14 (9.8)	68 (31.5)	39 (23.1)	13 (30.9)	26 (20.3)	160 (22.9)
**Has treated COVID-19 patients**	101 (33.7)	232 (83.7)	146 (47.1)	144 (46.7)	82 (27.3)	705 (47.0)

N – number of observations

*Number of observations (percentage).

†Counts and percentages do not add up to the total due to multi-response.

Most HCPs across the five countries (77.5%) reported that their workplace had COVID-19 guidelines and policies, but less than half reported receiving any training about the natural course of the disease (37.4%), management and treatment of COVID-19 (33.9%), how vaccines work (29.3%), and managing vaccination programs (22.9%). More than half of the participants (56.2%) reported availability of COVID-19 testing for their patients and reported that the available testing was free (76.2%) except for Tanzania where only 36% reported availability of free testing. Across all countries, 46.6% of the participants reported ever being tested for COVID-19 (with only 13.5% in Tanzania reporting being tested) and 23% reported testing positive for COVID-19. Across all countries, 470 of HCPs reported having treated COVID-19 patients, ranging from 83.7% in Ethiopia to 27.3% in Ghana (**Table 2**).

### Mental health and social stigma of health care providers during the COVID-19 pandemic

Across all countries, 9.4% of HCPs experienced mild psychological distress, 3.9% experienced moderate psychological distress, and 1.4% experienced severe psychological distress, with higher rates of mild psychological distress in Ghana (20.7%) compared to the other four countries. In the five countries overall, 7.8% of HCPs had a high anxiety score and 6.0% had a high depression score, but in Ghana, 19.1% and 15.4% had high anxiety and depression scores, respectively (**Table 3**). Across all countries, 13.8%, 3.1%, and 4.4% of HCPs reported social avoidance or rejection, denial of services, and physical violence due to their profession in the last six months, respectively. Higher proportions of HCPs reported social avoidance or rejection in Nigeria (18.3%) and Ghana (17.7%), as well as physical violence (9.6%) in Nigeria and (7.3%) in Ghana. Across all countries, nearly 48% of HCPs reported receiving congratulations or acknowledgement due to their profession in the last 6 months.

**Table 3:** Mental health and perceived stigma among health care providers in a phone-based survey in five sub-Saharan African countries, 2021*

	Burkina Faso	Ethiopia	Nigeria	Tanzania	Ghana	
	**Ouagadougou**	**Addis Ababa**	**Ibadan and Lagos**	**Dar es Salaam**	**Kintampo**	**Total**
**Number of health care providers**	300	277	312	310	300	1499
**Mental health outcomes**						
Mild psychological distress†‡	19 (6.3)	23 (8.3)	25 (8.0)	12 (3.9)	62 (20.7)	141 (9.4)
Moderate psychological distress†‡	3 (1.0)	13 (4.8)	12 (3.9)	2 (0.7)	28 (9.4)	58 (3.9)
Severe psychological distress†‡	2 (0.7)	4 (1.5)	3 (1.0)	0 (0)	12 (4.0)	21 (1.4)
High anxiety score‡§	6 (2.0)	19 (7.0)	29 (9.3)	6 (1.9)	57 (19.1)	117 (7.8)
High depression score‡§	7 (2.3)	25 (9.2)	11 (3.5)	0 (0)	46 (15.4)	89 (6.0)
**Perceived stigma**						
Social avoidance or rejection	19 (6.3)	31 (11.2)	57 (18.3)	47 (15.2)	53 (17.7)	207 (13.8)
Denial of health care, education, housing, or employment	2 (0.7)	13 (4.7)	12 (3.9)	5 (1.6)	14 (4.7)	46 (3.1)
Physical violence	5 (1.7)	6 (2.2)	30 (9.6)	3 (1.0)	22 (7.3)	66 (4.4)
Received Acknowledgment	103 (34.3)	79 (28.5)	213 (68.3)	97 (31.3)	223 (74.6)	715 (47.7)

N – number of observations

*Number of observations (percentage).

†Psychological distress was measured using the four-item Patient Health Questionnaire for Depression and Anxiety Scale (PHQ-4). Each item assessed whether the participant had experienced problems related to depression or anxiety over the past two weeks and had a numeric score ranging from 0 for “Not at all,” 1 for “Several days,” 2 for “More than half the days,” and 3 for “Nearly every day.” A total score was computed by adding up the scores of the four items, with a range of 0-12. The total score of psychological distress was categorized into none (total score = 0-2), mild (total score = 3-5), moderate (total score = 6-8), and severe (total score = 9-12).

‡one observation in Ethiopia and Nigeria is missing.

§An anxiety subscale (range = 0-6) and a depression subscale (range = 0-6) were created using the scores of the first and last two PHQ-4 questions. A subscale score of 3 or greater was considered a high level of anxiety and depression.

### Interruption of health service provision during the COVID-19 pandemic

Across all countries, most HCPs reported no interruption to childhood immunization (71.3%), vitamin A supplementation (75.9%), and malnutrition management (76.6%) due to the COVID-19 pandemic at the time of the survey. Across all countries, the mean aggregated interruption score was 0.71, 29.8% of HCPs reported a high interruption score (above the cut-off point of 0.71) for child health services. The majority of HCPs reported no interruption to antenatal care for pregnant women (74.6%), iron and folic acid for pregnant women (76.5%), and family planning and reproductive health (77.6%). Across all countries, the mean aggregated interruption score for maternal and reproductive health services was 0.68, with 26.8% reporting a high interruption score (above the cut-off of 0.68). For HIV treatment services, 75.3% of countries reported no interruption and 24.0% reported partial interruption, with higher percentages of reported partial interruption in Ethiopia (36.7%), Ghana (34.1%), and Nigeria (28.6%). For surgeries, 72.7% of countries reported no interruption and 26.7% reported partial interruption, with higher percentages of reported partial interruption in Ethiopia (44.6%), Ghana (37.2%), and Nigeria (35.6%). The overall percentage of health workers reporting high interruption scores was 26.9% (above the cut-off point of 1.81) and was 45.0%, 39.9%, 30.5%, 21.2%, 1.6% and in Ethiopia, Ghana, Nigeria, Burkina Faso, and Tanzania, respectively. This proportion was significantly different across sites (ANOVA F-statistic = 46.64, *P* < 0.000) (**Table 4**).

**Table 4:** Routine health care service provision during the COVID-19 pandemic from the perspective of health care providers in a phone-based survey from 2021*

	Serviced provided	Range of score	Burkina Faso (N = 300)	Ethiopia (N = 272)	Nigeria (N = 310)	Tanzania (N = 308)	Ghana (N = 299)	Total (N = 1489)
**Child health (CH)**	**Childhood immunization (N)†**	0-2	217	192	224	248	233	1114
	No interruption		155 (71.4)	119 (62.0)	150 (67.0)	243 (98.0)	127 (54.5)	794 (71.3)
	Partial interruption		61(28.1)	67 (34.9)	73 (32.6)	4 (1.6)	103(44.2)	308 (27.6)
	Complete interruption		1 (0.5)	3 (3.1)	1 (0.4)	1 (0.4)	3 (1.3)	12 (1.1)
	Missing/not applicable (N)		83	85	88	62	67	385
	**Vitamin A supplementation (N) †**	0-2	241	176	215	248	220	918
	No interruption		47 (79.7)	113 (64.2)	152 (70.7)	244 (98.4)	141 (64.1)	697 (75.9)
	Partial interruption		12 (20.3)	52 (29.6)	63 (29.3)	4 (1.6)	78 (35.5)	209 (22.8)
	Complete interruption		0 (0)	11 (6.2)	0 (0)	0 (0)	1 (0.4)	12 (1.3)
	Missing/not applicable (N)		241	100	97	62	80	581
	**Malnutrition management (N)†**	0-2	212	171	208	244	218	1053
	No interruption		161 (75.9)	110 (64.3)	149 (71.6)	242 (99.2)	145 (66.5)	807 (76.6)
	Partial interruption		51 (24.1)	56 (32.8)	58 (27.9)	2 (0.8)	72 (33.0)	239 (22.7)
	Complete interruption		0 (0)	4 (2.9)	1 (0.5)	0 (0)	1 (0.5)	7 (0.7)
	Missing/not applicable (N)		88	106	104	66	82	446
**Aggregated mean CH interruption score‡ (standard deviation)**		0-6	0.56 (0.9)	1.08 (1.5)	0.85 (1.3)	0.05 (0.3)	1.06 (1.3)	0.71 (1.2)
**Maternal and reproductive health (MH)**	**Antenatal care for pregnant women (N)†**	0-2	233	209	236	246	258	1182
	No interruption		182 (78.1)	127 (60.8)	163 (69.1)	244 (99.2)	166 (64.3)	882 (74.6)
	Partial interruption		50 (21.5)	77 (36.8)	71 (30.1)	2 (0.8)	91 (35.3)	291 (24.6)
	Complete interruption		1 (0.3)	5 (2.4)	2 (0.8)	0 (0)	1 (0.4)	9 (0.8)
	Missing/not applicable (N)		67	68	76	64	42	317
	**Iron and folic acid for pregnant women (N)†**	0-2	230	203	229	245	253	1160
	No interruption		182 (79.1)	130 (64.0)	161 (70.3)	243 (99.2)	172 (68.0)	888 (76.5)
	Partial interruption		46 (20.0)	69 (34.0)	67 (29.3)	2 (0.8)	80 (31.6)	264 (22.8)
	Complete interruption		2 (0.9)	4 (2.0)	1 (0.4)	0 (0)	.1 (0.4)	8 (0.7)
	Missing/not applicable		70	74	83	65	47	339
	**Family planning and reproductive health (N)†**	0-2	243	210	233	244	191	1121
	No interruption		196 (80.7)	133 (63.3)	169 (72.5)	243 (99.6)	129 (67.5)	870 (77.6)
	Partial interruption		46 (18.9)	73 (34.8)	63 (27.0)	1 (0.4)	62 (32.5)	245 (21.7)
	Complete interruption		1 (0.4)	4 (1.9)	1 (0.5)	0 (0)	0 (0)	6 (0.5)
	Missing/not applicable (N)		57	67	79	66	109	378
**Aggregated mean MH interruption score (standard deviation)‡**		0-6	0.59 (1.2)	1.11 (1.5)	0.85 (1.3)	0.02 (0.2)	0.88 (1.2)	0.68 (1.2)
**Other health service (OH)**	**HIV treatment services (N)†**	0-2	210	210	199	244	211	1074
	No interruption		158 (75.2)	128 (60.9)	141 (70.9)	243 (99.6	139 (65.9)	809 (75.3)
	Partial interruption		51 (24.3)	77 (36.7)	57 (28.6)	1 (0.4)	72 (34.1)	258 (24.0)
	Complete interruption		1 (0.5)	5 (2.4)	1 (0.5)	0 (0)	0(0)	7 (0.7)
	Missing/not applicable (N)		90	66	113	66	89	425
	**Surgeries (N)†**	0-2	235	211	238	272	218	1174
	No interruption		185 (78.7)	111 (52.6)	153 (64.3)	267 (98.2)	137 (62.8)	853 (72.7)
	Partial interruption		50 (21.3)	94 (44.6)	84 (35.3)	5 (1.8)	81 (37.2)	314 (26.7)
	Complete interruption		0(0)	6 (2.8)	1 (0.4)	0 (0)	0(0)	7 (0.6)
	Missing/not applicable		65	66	74	38	82	325
	**Tuberculosis treatment services and outpatient visits (N)†**	0-2	205	196	201	225	199	1026
	No interruption		151 (73.7)	128 (65.3)	142 (70.6)	224 (99.6)	127 (63.8)	772 (75.2)
	Partial interruption		54 (26.3)	67 (34.2)	57 (28.4)	1 (0.4)	69 (34.7)	248 (24.2)
	Complete interruption		0 (0)	1 (0.5)	2 (1.0)	0 (0)	3 (1.5)	6 (0.6)
	Missing/not applicable		95	81	111	85	101	473
**Aggregated mean OH interruption score (standard deviation)‡**		0-6	0.62 (1.2)	1.08 (1.3)	0.81 (1.2)	0.02 (0.2)	0.91 (1.2)	0.67 (1.2)
**Total health service interruption score**	**Aggregated mean total service interruption score (standard deviation)§**	0-18	1.56 (3.06)	3.0 (3.80)	2.2 (3.46)	0.08 (0.62)	2.50 (3.23)	1.81 (3.17)
	**High interruption (N, percentage)**		59 (21.2%)	109 (45.0%)	85 (30.5%)	5 (1.6%)	116 (39.9%)	374 (26.9%)

*Number of observations (percentage).

†Number of health care providers with non-missing data for each question.

‡The aggregated score for each health service was computed by summing responses for each specific health service included in each category. Responses were scored as 0 if there was no interruption (no impact on services), 1 for partial interruption (services operating at a minimum level/emergency services provided only), and 2 for complete interruption (services are paused). Missing responses and those who refused to answer, or did not know/don’t work, not applicable were excluded from the analysis.

§The total aggregated health service interruption score was computed by summing responses over the nine essential health services assessed in the questionnaire.

¶The high interruption score was defined as high when it was above the average aggregated score across all sites.

### Factors associated with essential health service interruption due to COVID-19

In adjusted models, HCPs in Ethiopia, Nigeria, and Ghana were 2.10 (ARR = 2.10, 95% CI = 1.59-2.74), 1.65 (ARR = 1.65, 95% CI = 1.25-2.17), and 2.61 (ARR = 2.61, 95% CI = 1.94-3.52) times as likely to report high service interruption, respectively, compared to Burkina Faso (**Table 5**). With each one-year increase in HCP age, the likelihood of reporting health service disruption increased by 1% (ARR = 1.01, 95% CI = 1.00-1.02). Nurses and other health professionals were 0.69 times more likely than doctors to report high service interruption (ARR = 0.69, 95% CI = 0.56-0.84). HCPs at health facilities with COVID-19 testing capability had a 41% increased risk (ARR = 1.41, 95% CI = 1.14-1.74) of reporting high service interruption scores. HCPs who worked at facilities that had workplace guidelines on COVID-19 had a 27% lower risk (ARR = 0.63, 95% CI = 0.53-0.77) of reporting high service interruptions, compared to those working at facilities that did not have workplace guidelines. HCPs who had ever been tested for COVID-19 had a 28% lower risk (ARR = 0.82, 95% CI = 0.69-0.97) in the likelihood of reporting high service interruption compared to those who had been tested (**Table 5**).

**Table 5:** Factors associated with high service interruption during the COVID-19 pandemic in five sub-Saharan African countries based on health care provider’s characteristics (N = 1088)*

Characteristics	N (%)	CRR	95% CI	*P*-value	ARR	95% CI	*P*-value
**Country**							
Burkina Faso	59 (16.0)	Ref			Ref		
Ethiopia	109 (29.5)	2.12	1.63-2.78	<0.01†	2.10	1.59-2.74	0.00‡
Nigeria	85 (23.0)	1.43	1.08-1.91	0.01†	1.65	1.25-2.17	0.00‡
Ghana	116 (31.4)	1.88	1.44-2.45	0.000†	2.61	1.94-3.52	0.00‡
**Age (mean, N)**	35.9, 364	0.99	0.99-1.00	0.73	1.01	1.00-1.02	0.02†
**Occupation**							
Doctors	116 (31.4)	Ref			Ref		
Nurses	253 (68.6)	0.77	0.65-0.92	<0.01†	0.68	0.56-0.84	0.00***
**COVID-19 testing availability**							
No	147 (31.2)	Ref			Ref		
Yes	254 (68.8)	1.36	1.13-1.64	0.001†	1.40	1.14-1.74	0.00†
**Ever tested for COVID-19**							
No	171 (46.3)	Ref			Ref		
Yes	198 (53.7)	0.94	0.79-1.10	0.4\	0.82	0.69-0.97	0.03†
**Workplace guidelines**							
No	91 (24.7)	Ref			Ref		
Yes	278 (75.3)	0.77	0.64-0.93	0.009†	0.63	0.53-0.77	0.000†
**Treated COVID-19 patient**							
No	176 (47.7)	Ref			Ref		
Yes	193 (52.3)	1.23	1.04-1.40	0.01†	1.09	0.89-1.31	0.06

N – number of observations, N (%) – number of observations (percentage), ARR – adjusted risk ratio, CI – confidence interval, CRR – crude risk ratio, ref – reference

*Risk ratios were calculated using modified Poisson regression. Tanzania was excluded from all models due to a low proportion (1.7%) of HCPs reporting service interruption to enable model convergence. Each model was adjusted for gender, facility, mild psychological stress concern for COVID-19 spread, and all other variables in the table.

†Significance set at 5%.

‡Significance set at 1%.

## DISCUSSION

Overall, HCPs reported that the COVID-19 pandemic resulted in disruptions across nine essential health services during the time of the survey (from July to December 2021), especially in Ethiopia, Ghana, and Nigeria. HCPs’ country, age, COVID-19 testing availability at the workplace, if the HCP had ever been tested for COVID-19, and presence of COVID-19 guidelines in the workplace were all significantly associated with health service disruption. A considerable proportion of HCPs also reported mild psychological distress, social avoidance or rejection, and physical violence due to their profession, especially in Ghana.

We found that almost all HCPs in our sample were concerned about COVID-19 and perceived a high risk of infection from COVID-19. They also reported high levels of prevention measures utilized in the workplace including masks, handwashing, and sanitizers. Most HCPs reported utilizing PPE and social distancing between patients as preventive measures. Compared to our previous survey a year before [10], the utilization of social distancing and PPE has decreased, but the proportion of HCPs who were concerned and perceived a high risk of infection appears to have increased. This difference may be due to the emergence and introduction of COVID-19 vaccines since the last survey round, which could cause a reduction in the practice of other COVID-19 preventive measures [27].

At the time of the survey (July – December 2021), 26.7% of HCPs reported a high service interruption score across nine essential health services, with considerable differences across sites. Tanzania had the lowest reported level of service interruption and Ethiopia and Ghana had the highest level. For each health service, most interruptions reported were partial rather than complete. This finding is consistent with a recent WHO report indicating that more than 34% of countries worldwide reported continued disruptions in over half of health services during the COVID-19 pandemic [28]. The most common reason for these disruptions was related to HCP workforce challenges including increased absenteeism, fatigue, shortages of HCPs, and competing needs from COVID-19 responses. Other reasons included shortages due to medical supply chain disruption and financial issues.

In the round one survey, the proportion of HCPs reporting high service disruption was 56% across Burkina Faso, Ethiopia, and Nigeria [13]. Similar studies in other SSA countries have found similar patterns of disruption, especially in maternal and child health [29,30]. Although the percentage of reported interruption has decreased since the round one survey, interruption in essential health services will likely have significant impacts on population health in the long term, especially in resource-limited countries. This interruption may severely upset economically marginalized groups and lead to an increase in health inequities [13,31,32].

We found that HCPs from Ethiopia, Nigeria, and Ghana were more likely to report high service disruption compared to those from Burkina Faso, which may be due to COVID-19 mitigation measures and caseloads in each country. These findings are in line with the Our World in Data COVID-19 government stringency index, which is a composite indicator intended to provide a measure of school closures, workplace closures, and travel bans due to COVID-19 across countries [33]. For example, at the start of this round two survey in July-August 2021, Burkina Faso had the lowest stringency index of 13 out of 100, followed by Tanzania (16.7), Nigeria (56.5), Ethiopia (43.5), and Ghana (42.6).

In this study, HCPs who had ever been tested for COVID-19 were less likely to report high service disruption than those who had not been tested. The advantages of increased testing coverage for COVID-19 are well-established and can lead to detection and reduction in COVID-19 transmission [34-38]. However, HCPs in facilities with COVID-19 testing capability were more likely to report service disruption than their counterparts. The finding could be explained by the fact that facilities with testing capabilities may be located in areas most affected by COVID-19. To support HCPs and reduce service interruptions, increasing testing capability and vaccination coverage and uptake is essential in LMICs [39-42].

The prevalence of psychological distress, anxiety, and depression among HCPs in five SSA countries was fairly low in this sample and was comparable to the results from the round one survey completed in Ethiopia, Nigeria, and Burkina Faso from July to November 2020 [10]. Across all sites, HCPs in Ghana had the highest proportion of psychological distress, anxiety, and depression. Other studies from 2020 in Asia using the PHQ-4 and other methods have reported higher proportions (38%) of HCPs experiencing psychological distress [43,44]. Differences between this study and others could be explained by the rapidly shifting landscape of the pandemic at the time of conducting the study [45,46]. For example, on December 27, 2021, there was a decrease in weekly new cases in Nigeria, Tanzania, and Ethiopia, but a slight increase in Burkina Faso and a large increase in Ghana [47].

We also found a considerable proportion of HCPs reported experiencing social avoidance or rejection and physical violence. Reports from Ethiopia and other countries worldwide indicate stigma toward HCPs during the COVID-19 pandemic [48-50]. Physical violence against HCPs (especially female and/or nurse HCPs) has been reported in Mexico, Iraq, and Brazil [51-53], there is limited evidence from Africa. There is a need for messaging campaigns during health emergencies to acknowledge HCPs and reduce negative perceptions among the public that may result in the stigmatization of HCPs [50].

Two key strengths of this study are the inclusion of HCPs from five countries as a follow-up study to a baseline survey in 2020, which can help to track changes in mental health, perceptions, prevention measures, and health service disruption as the COVID-19 pandemic progresses, and the use of phone-based surveys to enable remote and rapid data collection during the COVID-19 crisis [16]. Some limitations are the opportunistic selection of the study sites and potential sampling, non-response, and desirability bias, which may affect the representativeness of the results to the broader national contexts. However, we increased representativeness by including countries in geographically distinct locations across SSA, which may increase the generalizability of our findings to similar contexts. Additionally, the self-report nature of the questionnaire may introduce bias, especially in the assessment of health service interruption. Our estimates could be underreported due to the HCPs’ lack of knowledge of a health service interruption or if the HCP was not working in that area. Lastly, previous baseline coverage of health services was not considered in this survey, and we have no way to quantify the coverage of services before the pandemic. In many LMIC settings, coverage may have already been interrupted before the pandemic [54].

## CONCLUSION

In conclusion, we show that despite some progress, a significant proportion of health services continue to be disrupted due to COVID-19 in Burkina Faso, Ethiopia, Nigeria, and Ghana. This service interruption continues to impose severe global health challenges. Even though we found considerable availability of COVID-19 testing and care in health facilities, there are disparities between sites. In addition, psychological stress, societal rejection, and violence continue to affect HCPs. Increasing the COVID-19 vaccination rate for HCPs and the general community with appropriate and timely health education and advocacy to support HCPs could help to mitigate some of the negative impacts of the COVID-19 pandemic. Equitable sharing of COVID-19 vaccines across countries is crucial, along with further studies to generate knowledge on determinants of COVID-19 vaccine hesitancy among HCPs and interventions to increase vaccine uptake and confidence and reduce stigma against HCPs in SSA. Strengthening health systems in SSA is also essential to help countries recover from the interruptions experienced during the COVID-19 pandemic and to plan for future pandemics.
